# Clearance of p16Ink4a-positive cells in a mouse transgenic model does not change β-cell mass and has limited effects on their proliferative capacity

**DOI:** 10.18632/aging.204483

**Published:** 2023-01-12

**Authors:** Nadine Bahour, Lucia Bleichmar, Cristian Abarca, Emeline Wilmann, Stephanie Sanjines, Cristina Aguayo-Mazzucato

**Affiliations:** 1 Joslin Diabetes Center, Harvard Medical School, Boston, MA 02215, USA

**Keywords:** beta cells, mass, proliferation, senolysis, senescence

## Abstract

Type 2 diabetes is partly characterized by decreased β-cell mass and function which have been linked to cellular senescence. Despite a low basal proliferative rate of adult β-cells, they can respond to growth stimuli, but this proliferative capacity decreases with age and correlates with increased expression of senescence effector, p16Ink4a. We hypothesized that selective deletion of p16Ink4a-positive cells would enhance the proliferative capacity of the remaining β-cells due to the elimination of the local senescence-associated secretory phenotype (SASP). We aimed to investigate the effects of p16Ink4a-positive cell removal on the mass and proliferative capacity of remaining β-cells using INK-ATTAC mice as a transgenic model of senolysis. Clearance of p16Ink4a positive subpopulation was tested in mice of different ages, males and females, and with two different insulin resistance models: high-fat diet (HFD) and insulin receptor antagonist (S961). Clearance of p16Ink4a-positive cells did not affect the overall β-cell mass. β-cell proliferative capacity negatively correlated with cellular senescence load and clearance of p16Ink4a positive cells in 1-year-old HFD mice improved β-cell function and increased proliferative capacity in a subset of animals. Single-cell sequencing revealed that the targeted p16Ink4a subpopulation of β-cells is non-proliferative and non-SASP producing whereas additional senescent subpopulations remained contributing to continued local SASP secretion. In conclusion, deletion of p16Ink4a cells did not negatively impact beta-cell mass and blood glucose under basal and HFD conditions and proliferation was restored in a subset of HFD mice opening further therapeutic targets in the treatment of diabetes.

## INTRODUCTION

Type 2 diabetes develops in response to over-nutrition and lack of physical activity in subjects with underlying genetic and acquired predisposition to insulin resistance and β-cell dysfunction. Over time, β-cell compensation for insulin resistance fails, resulting in a progressive decline in function [1–6] and mass [7]. The endocrine pancreas is a slow turnover tissue [8] and β-cell proliferation decreases with age [9]. This age-dependent decline in proliferation is partly due to an increase in senescence marker and mediator *p16^Ink4a^*, a cyclin-dependent kinase inhibitor [10, 11]; mice with an additional copy of *p16^Ink4a^* had a significant decrease in β-cell proliferative capacity [12–15].

Aging and senescence are related but not interchangeable terms. Cellular senescence is a stress response that occurs throughout the lifespan in which cells remain metabolically active with an altered phenotype. Senescent cells accumulate with aging, resulting in changes in structure and function that include irreversible growth arrest, resistance to apoptosis and alterations in gene expression [16]. Senescence also leads to the secretion of an array of cell-specific proteins known as the senescence-associated secretory phenotype (SASP) that can induce dysfunction and entry into senescence of surrounding, healthy cells [17].

Previously, we showed that with age and insulin resistance, β-cell senescence increased [18, 19] and senolysis (selective deletion of senescent cells) improved β-cell function, gene identity, and blood glucose levels [19]. Additionally, it has been shown that senolysis can preserve β-cells in a model of Type 1 Diabetes [20]. INK-ATTAC mice are a transgenic whole-body FLAG-tagged line that allows specific deletion of cells expressing *p16^Ink4a^* upon administration of B/B homodimerizer, a synthetic drug that causes dimerization and activation of the caspase 8 only in *p16^Ink4a^*-positive cells [21, 22]. However, whether the elimination of *p16^Ink4a^*-expressing beta cells negatively impacts β-cell mass is unknown and whether residual cell proliferation can be rescued by removing local SASP remains to be determined.

To this end, we set out to explore the effects of removing *p16^Ink4a+^* senescent cells on the proliferative capacity and mass of β-cells using INK-ATTAC mice as a transgenic model. We hypothesized that removal of this cell population would decrease overall β-cell mass and rescue the proliferative capacity of the remaining cells due to local SASP elimination.

## RESULTS

### Removal of *p16^Ink4a^*-expressing cells in non-metabolically challenged middle-aged mice had no effect on β-cell mass while proliferation inversely correlated with senescence load

**Figure 1:**
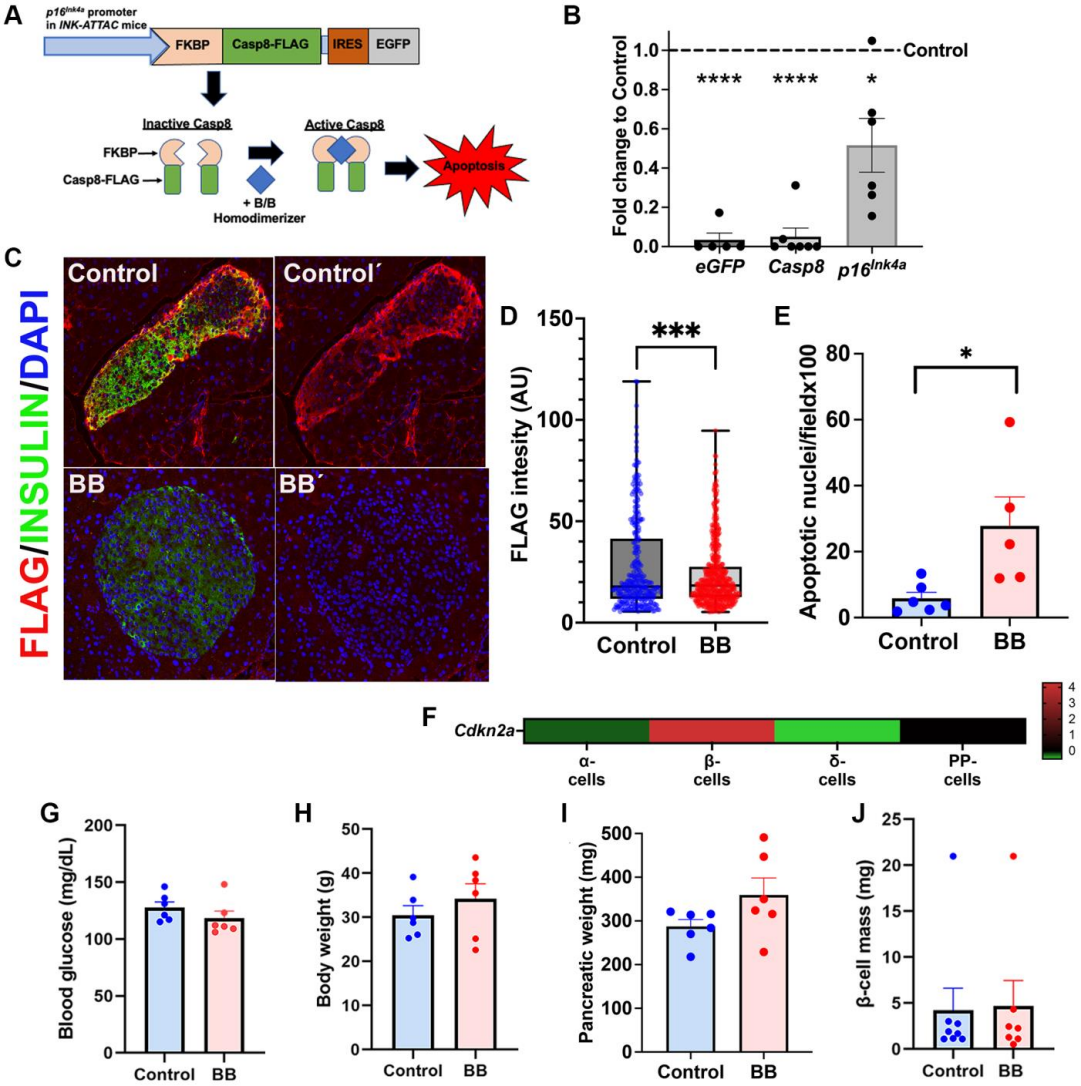
**INK ATTAC mice as a model to remove p16Ink4a^+^ cells.** (**A**) The INK ATTAC transgene is driven by the *p16^Ink4a^* promoter and encodes a Caspase 8 moiety that upon B/B homodimerizer administration leads to specific apoptosis of this cell subpopulation and is compared to animals treated with vehicle (referred to as controls). FLAG tag and eGFP expression can be tracked to measure effectiveness of deletion and are a surrogate marker of senescence load. Diagram modified from [21]. (**B**) qPCR from islets of animals treated with B/B homodimerizer and compared to control, untreated animals. A significant decrease of the transgene transcripts *Casp8* and *eGFP* and *p16^Ink4a^* was observed after treatment with B/B. (**C**) Representative confocal picture of islets from B/B treated and untreated animals showing a significant decrease of FLAG staining. (**D**) Quantification of FLAG intensity using image analysis software (Image J) showed a significant decrease in senescence load in islets from animals treated with B/B homodimerizer; n_control_ = 5 animals, 290 islets analyzed; n_treated_ = 8 animals, 518 islets analyzed mean+/−SEM, ^*^p = 0.0004 by unpaired *t*-test. (**E**) Increased number of apoptotic nuclei per field in pancreas from 8-month-old INK-ATTAC mice; n_control_ = 5 animals, 201 images analyzed; n_treated_ = 6 animals, 223 images analyzed; mean+/−SEM, ^*^p = 0.02 by unpaired *t*-test. (**F**) Heatmap of islet scRNASeq expression of *Cdkn2a* (encodes *p16^Ink4^*) in different islet cell types reveals enriched expression of *Cdkn2a* in β-cells. No significant changes of mean of (**G**) blood glucose levels (mg/dL), (**H**) body weight (g), (**I**) pancreatic weight (mg) of 8/9-month-old INK-ATTAC mice, *n* = 6 per group; and (**J**) Beta cell mass (mg) of 6-month-old INK-ATTAC mice, n_control_ = 7 and n_treated_ = 8.

INK ATTAC mice are a mouse transgenic model where *p16^Ink4a^* cells can be specifically tracked through FLAG staining and removed upon B/B homodimerizer injection (Figure 1A) [21]. Validation of this deletion model in pancreatic islets has been previously published [19] and showed that senescent islet cells were deleted after administration of two 3-day courses of B/B homodimerizer, 14 days apart, in 8–9 months old mice. FLAG immunostaining was used as a senescence marker and treatment with B/B homodimerizer significantly increased the proportion of FLAG-negative (non-senescent) islets [19]. Herein, islets from B/B homodimerizer treated animals were isolated and compared to islets from non-treated animals. A significant decrease of transgene markers *eGFP* and *Caspase8* were detected as well as a decrease in *p16^Ink4a^* mRNA (Figure 1B). It is interesting to note that *p16^Ink4a^* transcript levels decrease by 50% after B/B homodimerizer treatment whereas those of *eGfp* and *Caspase 8* are almost undetectable. This can be due to lower expression of second and third cistrons secondary to internal in-frame AUGs. This phenomenon has been described both in Drosophila [23] and mammalian cells [24]. In this model, FLAG intensity is an indicator of cellular senescence because it is driven by the p16^Ink4a^ promoter. Therefore, confocal pictures of FLAG-stained islets were taken using the same settings such that differences in intensity reflected differences in protein quantity. Quantification of FLAG intensity in insulin positive islets, showed a significant decrease in pancreas from B/B homodimerizer treated animals when compared to those treated with vehicle (Figure 1C and 1D) consistent with decreased senescence. Additionally, increased apoptotic nuclei were counted after TUNEL staining of pancreatic sections from animals treated with B/B homodimerizer (Figure 1E and Supplementary Figure 1). Since, INK-ATTAC mice are whole body transgenics and to understand the expression of p16^Ink4a^ in other islet cell-types, analysis of scRNASeq data from mouse islets was used and showed that β-cells are the main islet-cell population expressing *Cdkn2a*, the gene encoding p16^Ink4a^ (Figure 1F) both in control and insulin resistance conditions (Supplementary Figure 2). However, other islet cell types can also express the transgene to a lower extent. These results confirm that INK-ATTAC animals are a valid model for studying the effect of removing *p16^Ink4a^* expressing β-cells.

We used male and female 6–9-month-old INK-ATTAC mice fed a chow diet to evaluate the effects of B/B homodimerizer in animals without a metabolic challenge. Analysis revealed no changes in circulating blood glucose (Figure 1G), body weight (Figure 1H), pancreatic weight (Figure 1I), or β-cell mass (Figure 1J). Transcriptional analysis of insulin-sensitive peripheral tissues (fat, liver, red and white muscle) showed no significant differences in transcription of senescence genes *p16^Ink4a^* (encoded by *Cdkn2a*) (Supplementary Figure 3A) and *p21^Cip1^* (encoded by *Cdkn1a*) (Supplementary Figure 3B).

**Figure 2:**
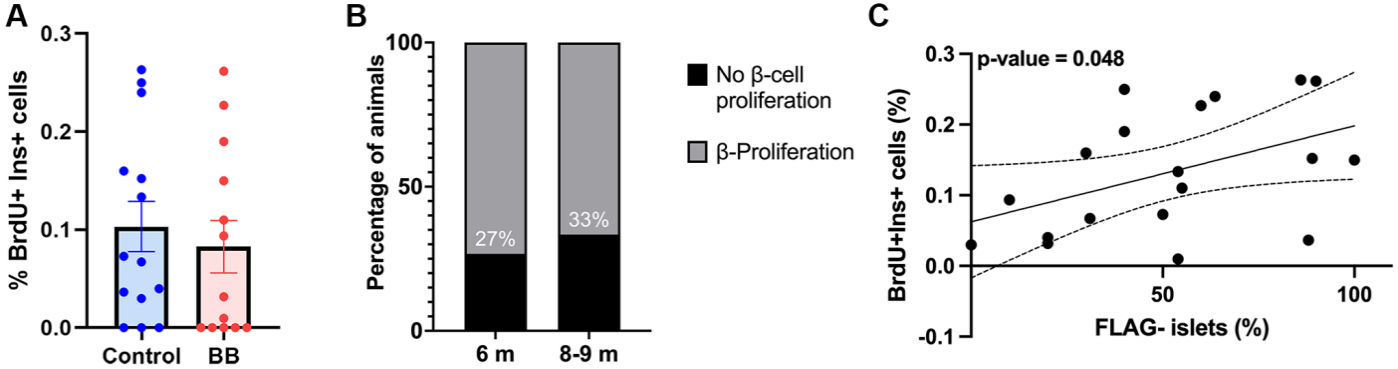
**Inverse correlation between cellular senescence load and proliferative capacity.** (**A**) Overall proliferation quantification of 6–9-month-old INK-ATTAC animals, n_control_ = 14, and n_treated_ = 13; (**B**) Percentage of islets with proliferating cells in 6 and 8/9-month-old INK-ATTAC mice measured by the percentage of BrDU^+^ Ins^+^ cells, n_6month_ = 15, and n_8–9month_ = 12; (**C**) Correlation between percentage of non-senescent FLAG^−^ islets (negative for FLAG staining) and proliferation (positive nuclear staining of BrDU) in 6–9-month-old (*n* = 19) INK-ATTAC mice. Line of best fit is shown along with dotted lines indicating 95% confidence intervals. *P*-value was calculated using the null hypothesis that the slope of the best-fit line equals 0.

To quantify the proliferation in the same cohort of middle-aged mice, pancreatic sections were stained for BrdU and insulin. B/B homodimerizer did not have an effect on β-cell proliferation at this age (Figure 2A). Consistent with the literature [13], there was a proportion of mice with undetectable proliferation levels, 26% at 6 months and 33% at 8–9 months (Figure 2B). To further assess remnant proliferation and its relation to cellular senescence, the percentage of animals that maintained proliferative capacity and its correlation to FLAG staining were quantified. A significant direct correlation was found between the proportion of non-senescent FLAG negative islets (negative for FLAG staining) and the percentage of proliferation (positive nuclear staining of BrDU) in 6–9-month-old animals (Figure 2C) supporting the idea that higher loads of senescent cells decrease β-cell basal proliferation while maintaining glucose homeostasis.

### Improved β-cell function with removal of *p16^Ink4a^* expressing cells in a HFD model in 1-year-old animals

**Figure 3:**
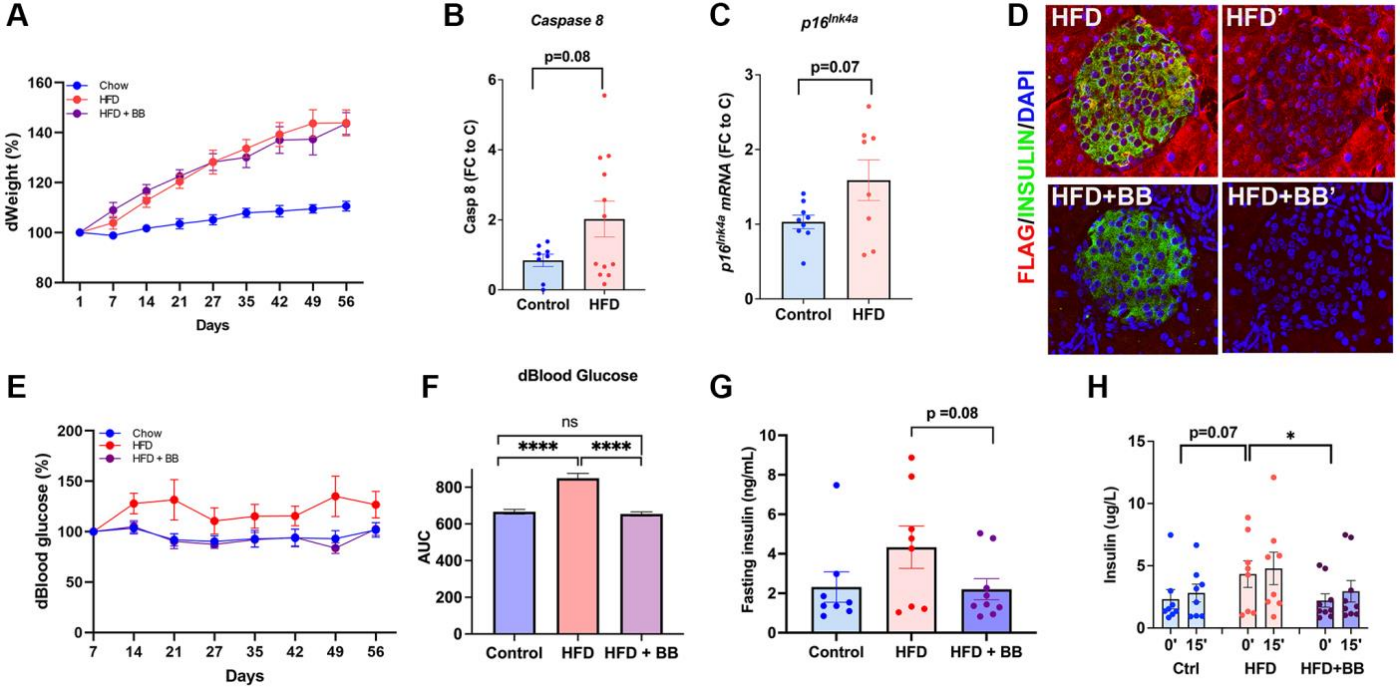
**Specific deletion of *p16^Ink4a^*-expressing cells improved metabolic profile.** (**A**) Bodyweight changes compared to the beginning of the HFD. (**B**) HFD increased transcription of *Caspase 8* by qPCR indicating acceleration of senescence in pancreatic islets. FC (fold change) to C (control). (**C**) HFD increased transcription of *p16Ink4a* by qPCR indicating acceleration of senescence in pancreatic islets. (**D**) Representative confocal images of HFD and HFD+ B/B homodimerizer treated islets. FC (fold change) to C (control). (**E**) Blood glucose percentage changes compared to the beginning of the treatment and (**F**) area under the curve for the three groups throughout the treatment period. (**G**) Fasting insulin (ng/mL) levels collected from tail blood during IPGTT (*p* = 0.08 by two-tailed unpaired *t*-test). (**H**) Glucose stimulated insulin secretion (GSIS) evaluated *in vivo* during IPGTT revealed restoration of basal insulin secretion after treatment with B/B homodimerizer. 1-year old INK-ATTAC mice; *n* = 8 control group, *n* = 8 HFD, *n* = 9 HFD = BB homodimerizer; males and females. ^*^p < 0.05 after *t*-test.

In adult animals, β-cell proliferation is induced by increased metabolic demand. Therefore, the effects of removing *p16^Ink4a^* cells in a high-fat diet (HFD) model were evaluated. One-year-old INK-ATTAC animals were maintained on a specific diet for 8 weeks in the following groups: control on a chow diet, HFD only, and HFD with four courses of B/B homodimerizer. Weekly body weight was monitored and showed a significant increase of 19% in both the HFD and HFD with B/B homodimerizer groups (Figure 3A). As previously reported [19], HFD accelerated β-cell senescence, which in this model was indicated as an increased *Caspase8* (Figure 3B) and *p16^Ink4a^* (Figure 3C) transcript in islets from animals fed a HFD. Representative confocal images of the pancreas from B/B treated animals showed a decrease in FLAG staining (Figure 3D) when compared with those treated with vehicle. HFD diet increased blood glucose by 27%, these levels were restored in the B/B treated group and comparable to chow animals (Figure 3E and 3F). Insulin tolerance test showed no significant differences among the groups (Supplementary Figure 4) even though results were different between males (Supplementary Figure 4A) and females (Supplementary Figure 4B). Fasting hyperinsulinemia was noted in the HFD group and restored after BB treatment (Figure 3G). Similarly, glucose stimulated insulin secretion (GSIS) *in vivo* during IPGTT, showed increased basal insulin in the HFD treated group which returned to normal after treatment with BB homodimerizer (Figure 3H). These results show that clearance of *p16^Ink4a^* in a HFD model partially restored glucose homeostasis.

### Removal of *p16^Ink4a^* expressing β-cells induced proliferation in a subset of HFD-treated adult mice

**Figure 4:**
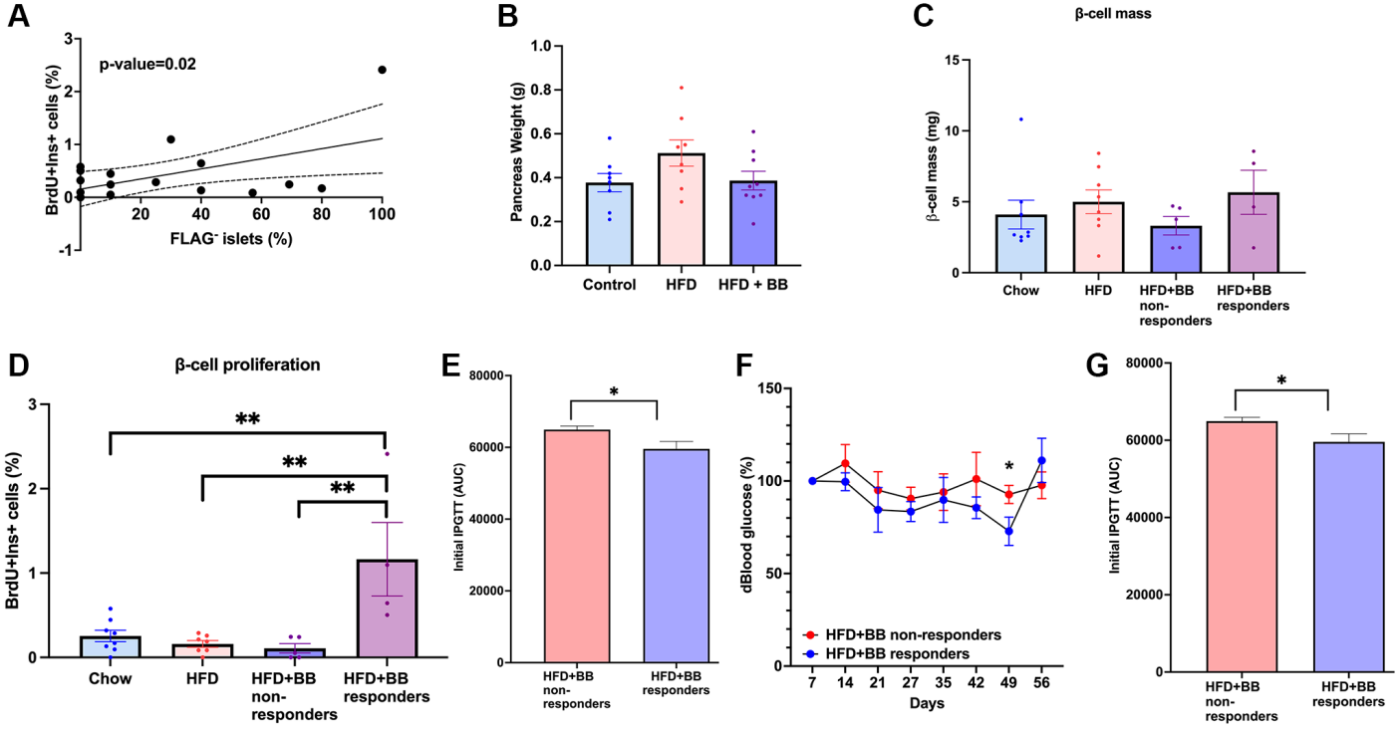
**Senolysis restored proliferative capacity in a subset of animals after a HFD metabolic challenge in 1-year old mice.** (**A**) Correlation between percentage of non-senescent FLAG^−^ islets and beta-cell proliferation in 12-month-old animals. Line of best fit is shown along with dotted lines indicating 95% confidence intervals. *P*-value was calculated using the null hypothesis that the slope of the best-fit line equals 0. (**B**) Pancreatic weight of the groups; (**C**) beta-cell mass calculated in (mg). (**D**) Beta-cell proliferation calculated by %BrDU^+^ Ins^+^ cells. Mean+/− SEM; significance calculated by ordinary one-way ANOVA with Tukey’s multiple comparisons. (**E**) Comparison between responders and non-responders to BB/homodimerizer treatment in the HFD group. AUC for IPGTT before treatment. (**F**) Blood glucose percentage changes compared to the beginning of the treatment and AUC (**G**) between responders and non-responders.

A significant correlation between the proliferation percentage and percentage of non-senescent β-cells was also seen at 12-months of age (Figure 4A) indicating that even at this age, removal of *p16^Ink4a+^* cells can have an impact on beta-cell proliferation.

There were no significant differences in pancreatic weight in different treatment groups (Figure 4B). Systematic imaging of pancreatic sections revealed no significant changes in β-cell mass amongst groups (Figure 4C) and overall, clearance of *p16^Ink4a^* expressing cells did not change the rate of β-cell proliferation. However, there was a subset of animals treated with B/B homodimerizer where BrdU incorporation was significantly increased and we termed this group “responders” which represented 44% (Figure 4D) of the total treated population. In the remainder 56% of the animals, proliferation did not increase in response to *p16^Ink4a^* clearance (Figure 4D). Further analysis comparing the non-responders to the responders revealed that the latter had better glucose clearance in the initial IPGTT (Figure 4E) and maintained overall lower blood glucose levels than non-responders throughout the experiment (Figure 4F and 4G). However, no differences were found between the two groups in the following parameters: gender, initial ITT, weight gain, fed or fasting insulin and fasting blood glucose (Supplementary Figure 5). These results suggest that responders had better β-cell function at the beginning of the experiment but further experiments are required to fully understand these differences.

### β-cell proliferation in a pharmacological model of insulin resistance

**Figure 5:**
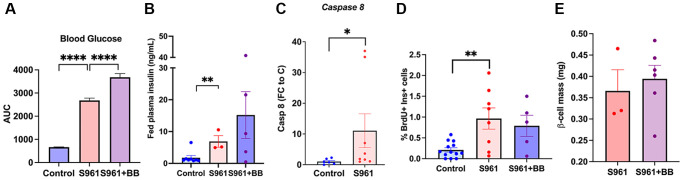
**Senolysis in an acute insulin resistance model, S961, did not affect beta-cell proliferative capacity.** (**A**) Area under the curve of blood glucose levels throughout the treatment period, n_control_ = 8, n_S961_ = 3, n_S961+BB_ = 5. (**B**) Fed plasma insulin (ng/mL) levels in control, S961, and S961 + BB; significance calculated by repeated unpaired *t*-test. (**C**) qPCR of islets from S961 treated animals increased *Caspase 8* transcription consistent with accelerated senescence. (**D**) Proliferation of beta cells (%) in 8–19-month-old mice. (**E**) Beta cell mass (mg) 18–19 month-old INK ATTAC mice, male and female. Mean +/− SEM, significance calculated by ordinary one-way ANOVA with Tukey’s multiple comparisons.

Insulin receptor antagonist S961 was used as an additional model to induce acute insulin resistance in 1.5-year-old male and female mice. S961 induced hyperglycemia (Figure 5A) and hyperinsulinemia (Figure 5B). S961 increased β-cell senescence as seen by an increase in *Caspase8* transcript (Figure 5C). Proliferation was significantly induced by S961 even in older animals (Figure 5D, Supplementary Figure 6A) however this proliferative rate was not further increased by deletion if *p16^Ink4a^* cells (Figure 5D, Supplementary Figure 6B) and no changes in beta cell mass were found after treatment (Figure 5E). Removal of *p16^Ink4a^* expressing cells in this group worsened blood glucose levels (Figure 5A) indicating that the functional reserve was adversely affected by the removal of this cell population.

### The targeted *p16^Ink4a^* subpopulation is non-proliferative and non-SASP producing

**Figure 6:**
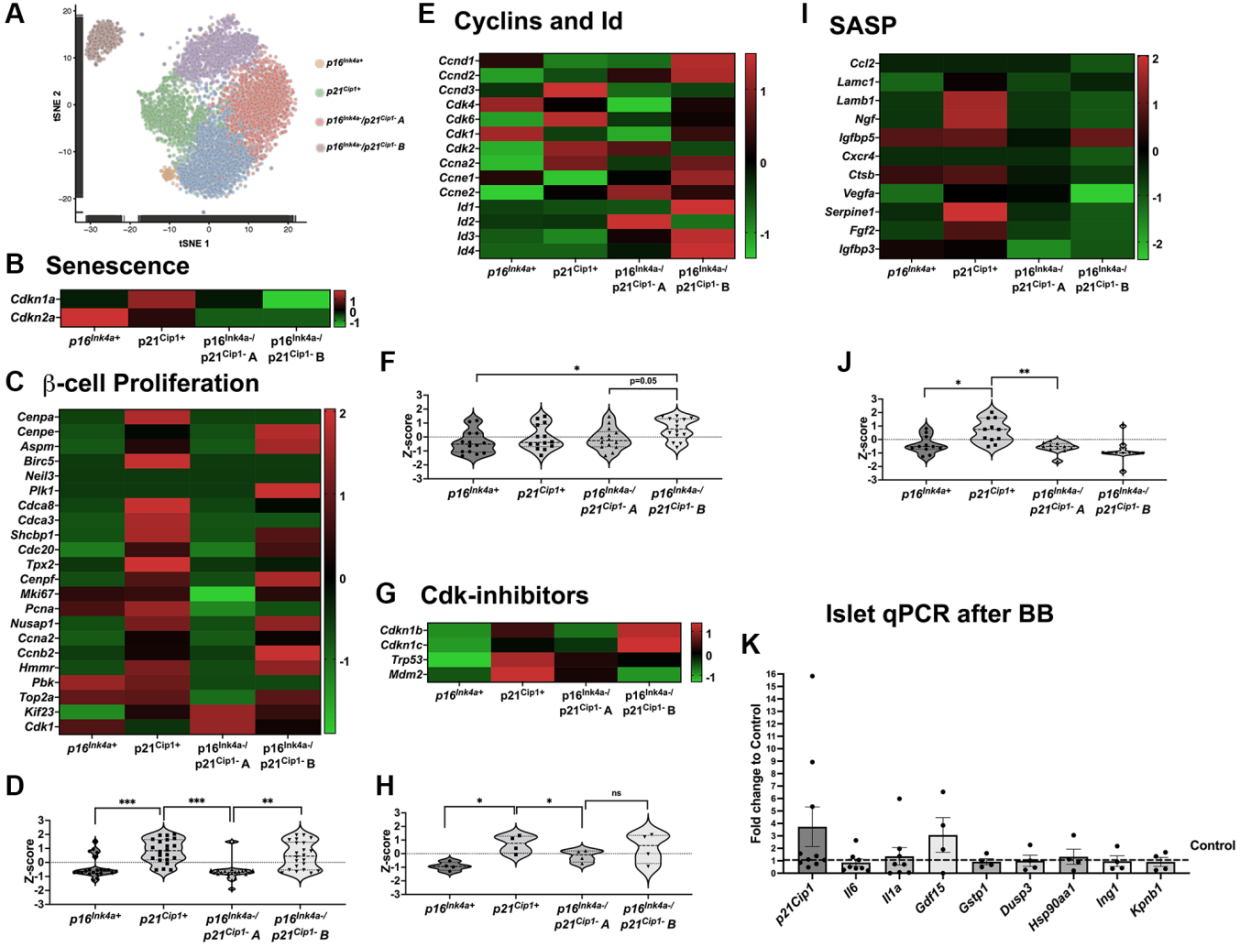
***p16^Ink4a^*-expressing cells do not proliferate or secrete SASP compared to other senescent cells.** (**A**) tSNE of beta-cell clusters based on *Ins2* expression and four subpopulations highlighted: p16^Ink4a+^, p21^Cip1+^ (senescent) and p16^Ink4a−^/p21^Cip1−^ A and p16^Ink4a−^/p21^Cip1−^ B (non-senescent). (**B**) Heat map showing beta cell senescence genes. (**C**, **D**) Heat map and violin plot showing beta-cell proliferation genes. (**E**, **F**). Heat map and violin plot showing beta-cell expression of cyclins and Id. (**G**, **H**) Heat map and violin plot of Cdk-inhibitors; (**I**, **J**) Heat map and violin plot of SASP genes. (**K**) qPCR of islets treated with B/B homodimerizer show no changes in the transcript of p21Cip1 and several transcription factors in the INK ATTAC model. Significance calculated by ordinary one-way ANOVA with Tukey’s multiple comparisons.

To further understand the lack of effects of the removal of *p16^Ink4a^* cells in β-cell proliferation, scRNASeq analysis was performed in previously reported β-cells pooled from control and S961 treated animals [25]. t-SNE analysis revealed β-cell subpopulations that were divided into 6 clusters (Figure 6A). Analysis was concentrated in 4 of these subpopulations (Figure 6B). Two senescent subpopulations: *p16Ink4a^+^*, *p21Cip1^+^*, and two non-senescent subpopulations: *p16Ink4a^−^/p21Cip1^−^* A and *p16Ink4a^−^/p21Cip1^−^* B. In the INK-ATTAC model, only the *p16Ink4a^+^* cells were removed (Figure 1B) while *p21Cip1^+^* remained unchanged (Figure 6K). scRNASeq allowed further analysis of the proliferative capacity and SASP transcription of these specific cell populations. As previously described, *p16Ink4a^+^* cells were non-proliferating (Figure 6C and 6D) whereas *p21Cip1^+^* and *p16Ink4a^−^/p21Cip1^−^* cells upregulated of proliferative genes (Figure 6C, 6D). We believe that the upregulation of proliferation genes in *p21Cip1^+^* represents a population of growth arrest during early senescence [26, 27]. This is further supported by significant upregulation of Cdk inhibitors (Figure 6G and 6H) in this subpopulation. Other cyclins and *Id* were significantly upregulated in the *p16Ink4a^−^/p21Cip1^−^ B* subpopulation (Figure 6E and 6F). scRNASeq data was further inquired for the transcriptome of SASP factors known to be upregulated in β-cells (Figure 6I and 6J). These changes were confirmed by performing qPCR on islets from BB homodimerizer treated animals. The main SASP-producing β-cell subpopulation was the *p21Cip1^+^* which persisted in the islets from mice treated with BB (Figure 6K).

These results highlight the existence of different senescence subpopulations of β-cells with varying proliferative and SASP producing characteristics which are differentially targeted in this transgenic model. In this particular case, the removal of *p16^Ink4a+^* cells did not result in the removal of SASP-producing subpopulations which might account for a lack of effects upon proliferative capacity.

In summary, a transgenic model that targets *p16^Ink4+^* senescent cells, did not alter β-cell mass and increased proliferation levels only in a subset of mice in response to HFD. This is potentially due to remaining senescent cells which are negative for *p16^Ink4a^* and whose local SASP production continues to impair the proliferative capacity of the remaining cells. Additionally, a direct correlation between β-cell proliferative capacity and non-senescent islet cells was found.

## DISCUSSION

Cellular senescence has been studied in the context of type 2 diabetes and shown to play a role in the progression of the disease. Deletion of senescent cells has been associated with improved glucose levels and β-cell function [19]. In this study, the effects of deleting *p16^Ink4a^*-expressing senescent cells on β-cell function, mass, and proliferative capacity were evaluated. Removal of this senescent subpopulation did not affect β-cell mass and increased proliferation only in a subset of animals. However, endogenous proliferative capacity is inversely correlated with β-cell senescence underscoring the relationship between them. scRNASeq revealed that the targeted *p16^Ink4a^* β-cell population was non-proliferative and not SASP producing implying that local SASP production remained and was responsible for the terminal cell cycle arrest of β-cells.

Given the known decrease in β-cell mass at the time of T2D diagnosis, pursuing interventions that eliminate a percentage of the remaining cells is concerning. Therefore, a crucial pending question when considering senolysis as a therapeutic option is whether the β-cell mass will be decreased and therefore worsen the development of the disease. Given that senolysis targets senescent cells, our previous reports have found that middle-aged mice (3–6 m) have less than 5% senescent β-cells whereas this increases to approximately 12% at 2-years of age as measured by β-Gal activity [18]. When considering insulin resistance models, S961-treated middle-aged mice had 10% β-Gal positive cells [19]. Put together, these results indicate that senolysis would target a range between 5–10% of the β-cell mass. The impact this would have on regulating blood glucose levels can be estimated by reports of the β-cell functional reserve which has been estimated between 20–25% in rats [28] and 50–70% in type 1 [29, 30] and type 2 [7] diabetes. In this study, blood glucose levels were maintained or improved after removal of *p16^Ink4a^* expressing cells in HFD and non-challenged models. However, in S961 treated animals, senolysis significantly increased hyperglycemia suggesting that in the setting of extreme insulin resistance, targeting senescent cells is counterproductive. The fact that there was a different proliferative response between the HFD and the S961 group when both were treated with B/B homodimerizer is intriguing. This is probably due to β-cell proliferation induced by S961 with no further proliferation with senolysis, however HFD by itself did not produce this increase in proliferation and was more amenable to the positive effects of senolytic interventions. Herein, we show that senolysis specifically targeted at *p16^Ink4a+^* positive cells does not affect the β-cell mass and does not worsen blood glucose levels under physiological conditions.

Lack of proliferative capacity and SASP secretion are two of the hallmarks of senescent cells [31]. Therefore, decreasing the load of senescent cells in a given tissue could hypothetically increase the proliferative capacity of the remaining cells due to the elimination of SASP. Our results show that *p16^Ink4a^*-directed senolysis did not change basal proliferative rates and was able to induce proliferation in response to HFD only in a subset of animals. These results are surprising given previous reports of decreased islet proliferation in *p16^Ink4a^* overexpression mouse models [11]. These contrasting outcomes might be due to cell-autonomous mechanisms in the deletion model that were induced by *p16^Ink4a^* cells and persisted in the remaining neighbouring cells. This concept is further supported by transgenic *p16^Ink4a^* overexpression demonstrating β-cell-autonomous effect of proliferative restriction [32].

Both *p21^Cip1^* and *p16^Ink4a^* are known markers and effectors of senescence. Analysis of scRNAseq data revealed the presence of different β-cell subpopulations with different levels of *p21^Cip^* and *p16^Ink4a^*. Interestingly, amongst the two, it was the *p21^Cip+^* subpopulation the one that transcribed most of the known β-cell SASP factors implying that these were not removed in the INK ATTAC transgenic model. These residual *p21^Cip+^* senescent and SASP producing cells could continue to impair the proliferative capacity of remaining cells and should be targeted before a claim of lack of effects of senolysis upon β-cell proliferation can be conclusively made. Whereas *p21^Cip1^*-null mice reported normal basal β-cell proliferation [33], it would be interesting to subject these animals to a metabolic challenge (HFD, pregnancy) and see whether proliferation is affected in those instances. Interestingly, an increased β-cell replication has been reported in multiple endocrine neoplasia type 1 syndrome associated with loss of *p18* and *p27* [34]. Additionally, it has been reported that *p27* loss is associated with increased β-cell proliferation and mass after HFD or leptin receptor loss [35, 36].

Whereas senolytic therapies are an approach to senescent cell elimination, they can have adverse effects that need to be considered. First, they are not cell-type specific, which can be a challenge for studying their effects on specific tissues, organs and diseases. Second, senolytic therapies can interfere with wound healing and have oncogenic effects [37]. Whereas translation of senolysis would employ senolytic drugs, the INK-ATTAC transgenic model provides a useful and targeted strategy to initially study the potential effects of removing a bona fide senescent subpopulation. An additional transgenic model that can be used to study the effects of senolysis is the *p16*-3MR [38] However, future studies should employ drugs to obtain a physiological perspective of its effects *in vivo*.

Further studies will also need to elucidate the effect of *p21^Cip1^* on β-cell proliferation as well as inhibiting SASP. Senomorphic drugs, which specifically inhibit SASP secretion, would be one option to minimize the effect of senescent cells on healthy, surrounding cells or senolysis specifically directed at this senescent subpopulation.

## METHODS

### Animals

All experiments were conducted at Joslin Diabetes Center with approval of its Animal Care and Use Committee. Mice were kept in a conventional facility in a 12-hour light/dark cycle with water and food *ad libitum* and a temperature between 22.2–22.7°C. When specified, a high-fat diet (HFD) 60kcal% fat (Research Diets; NJ, USA) was used for the specified amount of time. C57Bl6/J mice (Jackson Laboratory; ME, USA) were used for some experiments as specified. Breeding pairs of INK-ATTAC mice were a gift from Dr. Jan van Deursen [21] and all the animals used came from our colony. Middle-aged animals were ages 6–9 months with 15 animals 6-month-old (n_female_ = 7 and n_male_ = 8) and 12 animals 8–9-month-old (n_female_ = 6 and n_male_ = 6). 25 one-year-old animals were used for HFD (n_female_ = 13 and n_male_ = 12). 9 animals were used for S961 ages 18–19 months (n_female_ = 2 and n_male_ = 7). Animals of different ages were used because age is one of the main factors determining both beta-cell proliferation and senescence load. By analyzing animals of different ages, it is possible to evaluate whether the effects of *p16^Ink4a^* removal are dependent on chronological age.

### Assessment of glucose homeostasis

Bodyweight and morning fed glucose levels were monitored weekly. Blood glucose values were measured using a glucometer (Contour; NJ, USA) on blood from tail snip. For intraperitoneal glucose tolerance tests, blood samples for glucose levels from mice fasted for 6 hours were collected at 0, 15, 30, 60, 90, and 120 min after intraperitoneal injection of 10% glucose solution (Sigma Aldrich; MO, USA; 0.02 mL/g body weight). Insulin was measured from serum collected time of sacrifice using an insulin ELISA kit (Mercodia; NC, USA). For insulin tolerance tests, mice were fasted for 4 hours, insulin (Humulin R, Eli Lilly; IN, USA; 1 unit/g body weight) was injected intraperitoneally, and blood glucose was measured at 0, 15, 30, and 60 min.

### Senolytic treatment

The deletion protocol of *p16^Ink4a^*-expressing cells for INK-ATTAC mice consisted of the administration of 3-day courses of B/B homodimerizer (Takara Bio; CA, USA; 10 mg/kg) repeated every 14 days to activate the caspase-8 moiety. Mice were treated with an intraperitoneal injection of vehicle (Ethanol: polyethylene glycol 400: Tween 2% at 2:5:43) or B/B homodimerizer (in ethanol: polyethylene glycol 400: Tween 2% at 2:5:43).

### S961 treatment

S961 was a generous gift from Dr. Lauge Schaffer (Novo Nordisk; Denmark) [39]. Vehicle (PBS) or 20 nmol S961 was loaded into an Alzet osmotic pump and surgically implanted subcutaneously in the back of the anesthetized mice [40] and changed weekly for a total of two weeks.

### Pancreas isolation for quantification of mass and proliferation

Mice were injected with 10 mg/mL BrdU (Sigma Aldrich; MO, USA; 10 uL/g body weight) intraperitoneally, 6 hours before sacrifice. Under anesthesia, the pancreas was excised, weighed, and fixed in 4% (para)-formaldehyde (PFA) for 2 hours and embedded in paraffin for sectioning and immunostaining.

### Immunostaining and morphometric evaluation

Paraffin sections were deparaffinized with xylene and ethanol gradients, washed with PBS, permeabilized with Triton-X 0.3%, antigen retrieval was completed with heated citric acid for all stains and blocked with normal donkey serum. After washing with PBS and 2% lamb serum, slides were incubated overnight at 4°C with the primary antibody (Table 1). This was followed by one wash with PBS + 2% lamb serum and incubations for 1hr with secondary antibodies (Table 1). The slides were mounted with Fluoroshield + DAPI for nuclear localization (Sigma Aldrich; MO, USA).

**Table 1:** Antibodies.

**Antibody**	**Species**	**Manufacturer and item no.**	**Concentration**
Anti-Insulin	Guinea Pig	Abcam; ab195956	1:400
Anti-FLAG	Rabbit	R&D; MAB8529	1:250
Anti-FLAG	Mouse	Sigma Aldrich; F1804	1:500
Anti-BrdU	Mouse	Sigma Aldrich; B8434	1:50
488 anti-Guinea Pig IgG	Donkey	Jackson ImmunoResearch Laboratories; 706-545-148	1:200
594 anti-Rabbit IgG	Donkey	Jackson ImmunoResearch Laboratories; 711-585-152	1:200

For quantification, islet images were captured systematically covering the whole section in confocal mode on a Zeiss LSM 710 microscope. For mass quantification, the entire section was pictured using the tile-scan system and images were quantified using ImageJ (https://imagej.net/ij/index.html) and Adobe Photoshop (https://www.adobe.com/products/photoshop.html). Beta-cell mass was calculated by multiplying the relative area of beta cells by the pancreatic weight. For all other stains, islets were pictured, coded, and read blindly. For BrdU, 589–3295 cells were counted from at least 10 islets per animal. Quantification of FLAG staining was done using ImageJ and selecting the islet area through insulin staining.

### TUNEL stain and quantification

TUNEL staining was done using TUNEL Assay Kit-HRP-DAB (ab206386) from Abcam following their protocols. Positive staining, indicating apoptotic nuclei, was determined by a dark brown color (Supplementary Figure 1). Quantification was done using a brightfield microscope and Ocular^®^ Scientific Image Acquisition Software. For quantification, systematic alternating pictures were taken at 20× magnification of the whole tissue and positive nuclei were manually counted.

### Quantitative real-time PCR

RNA was extracted from cells using the RNEasy Plus Mini Kit (QIAGEN; Germany); SuperScript reverse transcriptase (Invitrogen; MA, USA) was used to reverse transcribe RNA and generate cDNA for quantitative PCRs (Table 2). To measure gene expression levels, we used Fast SYBR green (ThermoFisher; MA, USA) and ΔCT values to βActin were calculated.

**Table 2:** Primer sequences.

**Gene**	**Forward**	**Reverse**
βActin	ACCGTGAAAAGATGACCCAG	GTACGACCAGAGGCATACAG
Cdkn1a	GCAGATCCACAGCGATATCC	CAACTGCTCACTGTCCACGG
Cdkn2a	CCCAACGCCCCGAACT	GCAGAAGAGCTGCTACGTGAA

### Single-cell RNA-seq

Single-cell RNA-seq data presented in this article is an analysis of pooled beta-cells from islets from C57BL6/J mice treated with PBS and S961. Beta cells were identified as having high Ins2 expression and raw data was previously published [25] and deposited under accession number GSE149984. Briefly, islets from animals with S961 or PBS pumps (described above) were isolated from mice for scRNA-seq, cultured overnight, and dispersed. Transcriptomic analysis was performed using the 10× Genomics Chromium Single Cell Gene Expression Assay core at Brigham and Women's Hospital. The Illumina NextSeq500 was used for sequencing, and the two libraries were pooled evenly on one lane. Data analysis was performed by the Bioinformatics and Biostatistics Core at Joslin Diabetes Center. Raw sequencing data were demultiplexed, aligned to the mouse genome and UMIcollapsed using CellRanger [41]. The inclusion criteria were: UMI >500, detected genes >1000, and mitochondrial genes < 20%. Data were analyzed using R. Deconvolution of size factors from cell pools, estimation of technical noise, and denoised Principal Component Analysis (PCA) were done using scran [42, 43]. t-Distributed Stochastic Neighbor Embedding (t-SNE) plots were made using scatter [44]. Cells were clustered into putative subpopulations using a shared-nearest-neighbor graph constructed from the PCA coordinates, and the clusters were found using a spin-glass algorithm [45, 46]. High Ins2 expression was used to identify beta cell clusters. Differential gene expression was assessed using linear modeling with limma [47].

### Quantification and statistical analysis

Data are shown as mean ± SEM. For statistical analysis, unpaired Student’s *t*-tests were used to compare two groups and one-way ANOVA followed by post hoc test for more than two groups. A normality test was performed and when not passed non-parametric statistics were run. A *p*-value ≤ 0.05 was considered significant. Prism 9.0 software by GraphPad (https://www.graphpad.com/scientific-software/prism/) was used for graphs and statistical analysis (significance and distribution). Animals were assigned to either control, intervention, or treatment groups to have equal age and gender distribution among all groups. The B/B homodimerizer treatment was completely composed of animals positive for the INK ATTAC transgene while the intervention and control groups had a mixture of positive and negative animals. No differences have been shown between animals with and without the transgene. Animals were excluded from the analysis if they became sick or developed physical anomalies.

### Data availability

scRNASeq data reported in this article can be obtained with the accession number GSE149984.

## Supplementary Materials

Supplementary Figures
