# Supplementary material for: Clearance of p16Ink4a-positive cells in a mouse transgenic model does not change β-cell mass and has limited effects on their proliferative capacity
Source: Aging (Albany NY). 2023 Jan 12;15(2):441–58. doi: 10.18632/aging.204483 (PMC9925693; doi:10.18632/aging.204483)
Supplement: Supplementary Figures [file aging-15-204483-s001.pdf]

SUPPLEMENTARY FIGURES

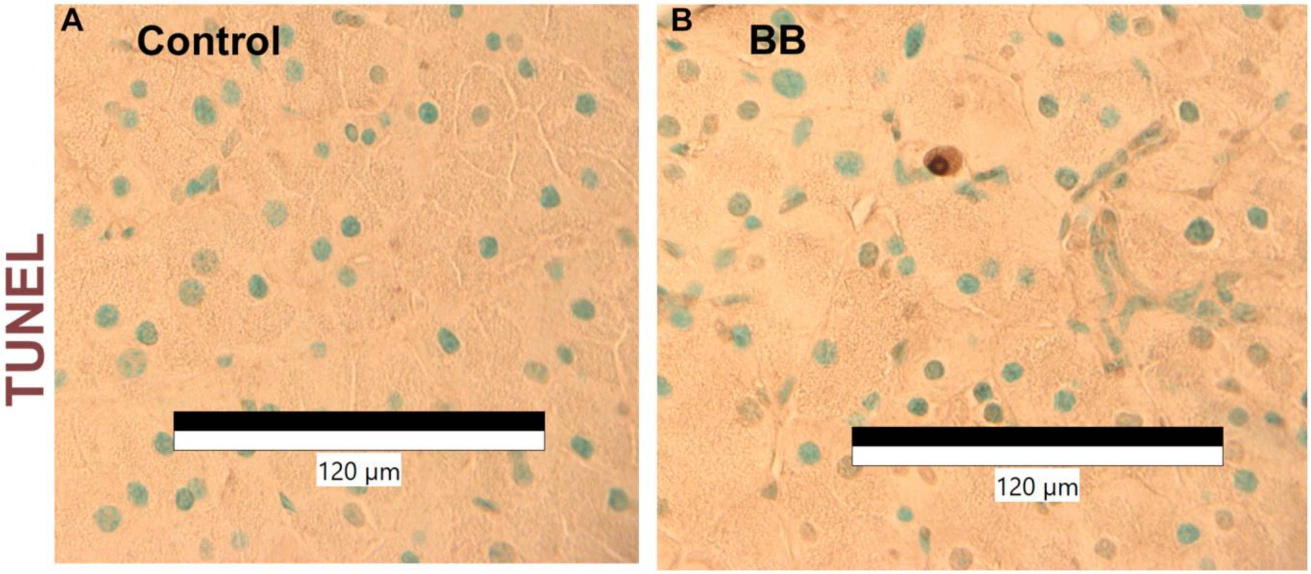

Supplementary Figure 1. (Related to Figure 1E): Representative pictures of apoptosis quantification in whole pancreatic sections from 8–9-month-old INK ATTAC mice treated with vehicle or with B/B homodimerizer to induce apoptosis of *p16^{Ink4a}* positive cells. Positive TUNEL appears as a nuclear brown staining with methyl green nuclear counterstain. (**A**) $n_{control}$ = 5 animals, 201 images analyzed; (**B**) $n_{treated}$ = 6 animals, 223 images analyzed.

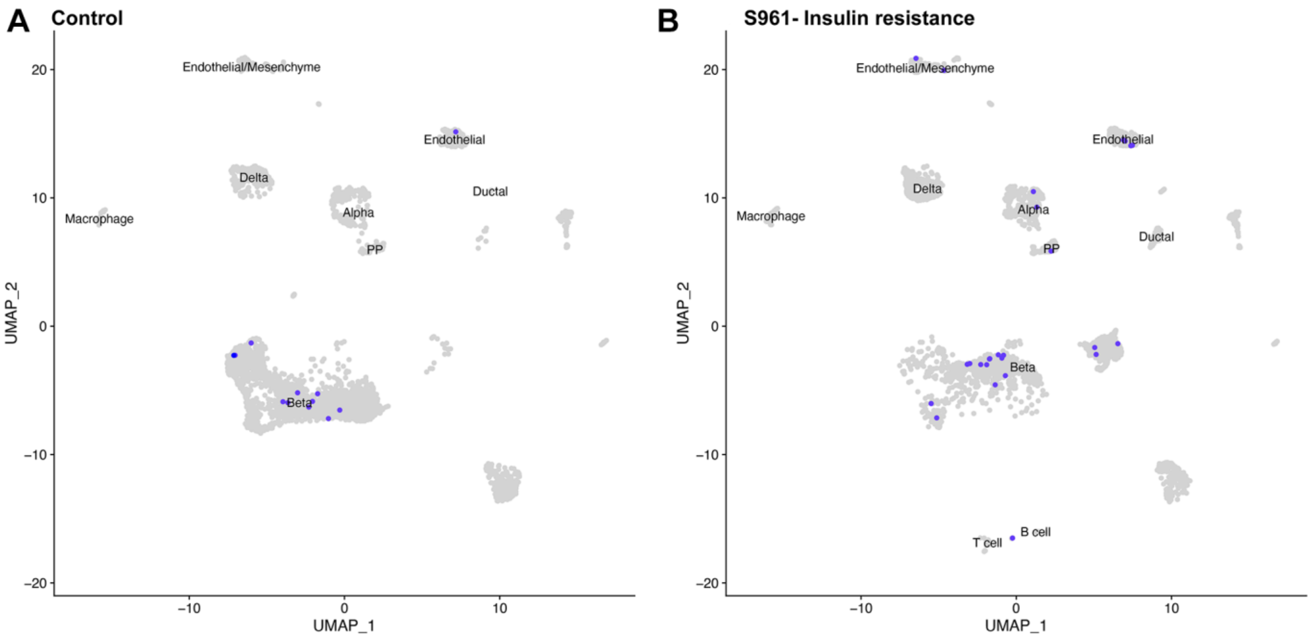

Supplementary Figure 2. (Related to Figure 1F): UMAP plot displaying the major islet-cell cluster of mouse pancreatic islets and expression of *Cdkn2a* in both control (**A**) and S961-insulin resistance (**B**) models.

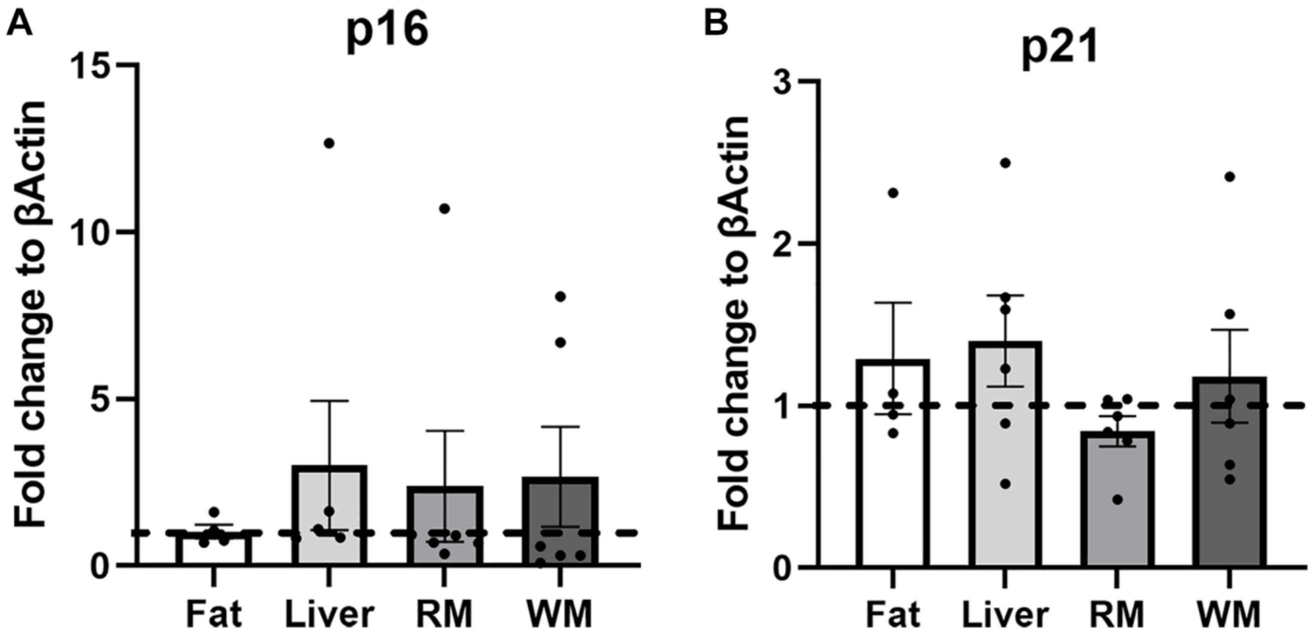

Supplementary Figure 3. (Related to Figure 1G–1J): (**A**, **B**) qPCR results from (**A**) *p16*^{Ink4a} and (**B**) *p21*^{Cip1} mRNA expression for fat, liver, white muscle (WM), and red muscle (RM).

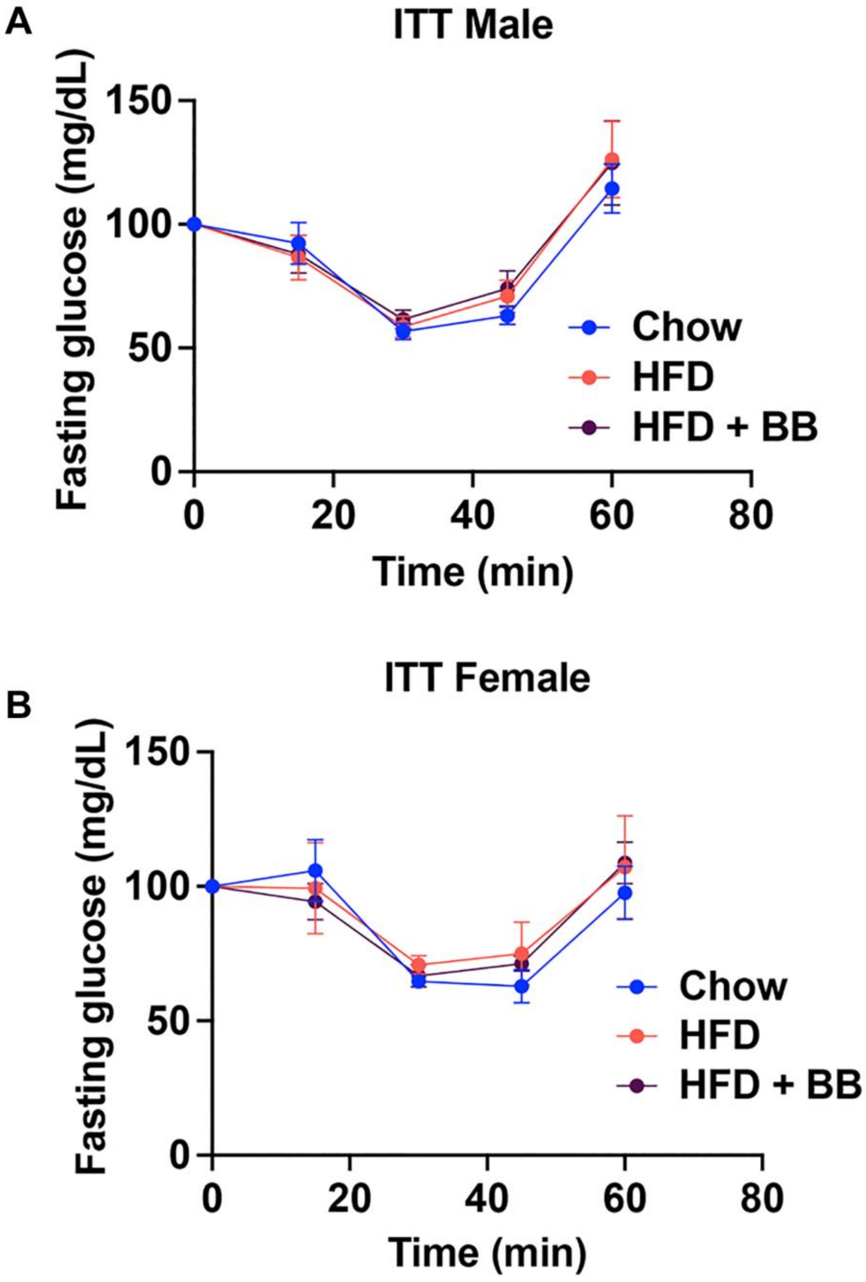

Supplementary Figure 4. (Related to Figure 3): Blood glucose (%) levels during ITT for 1-year-old mice with HFD-induced insulin resistance in male (**A**) and female (**B**) mice.

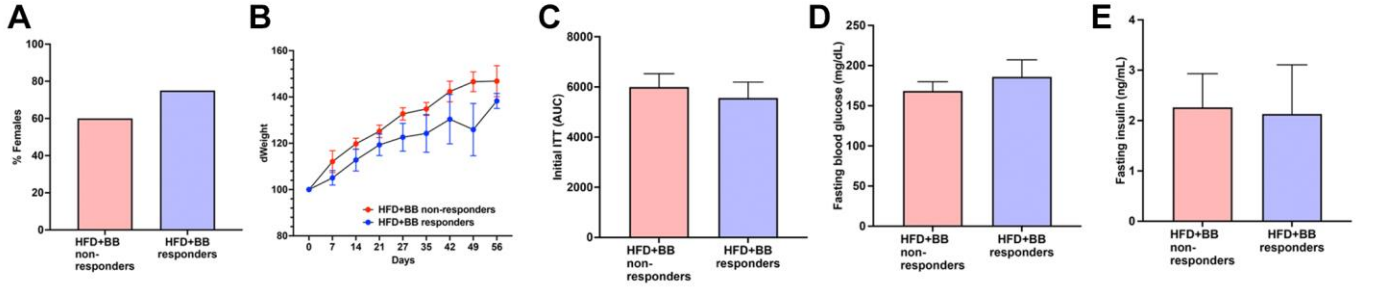

Supplementary Figure 5. (Related to Figure 4) The following physiological parameters where not different between HFD + BB homodimerizer responders and non-responders: gender (**A**), weight increase during study (**B**), initial glucose clearance during insulin tolerance test (**C**), fasting blood glucose (**D**) and fasting insulin (**E**).

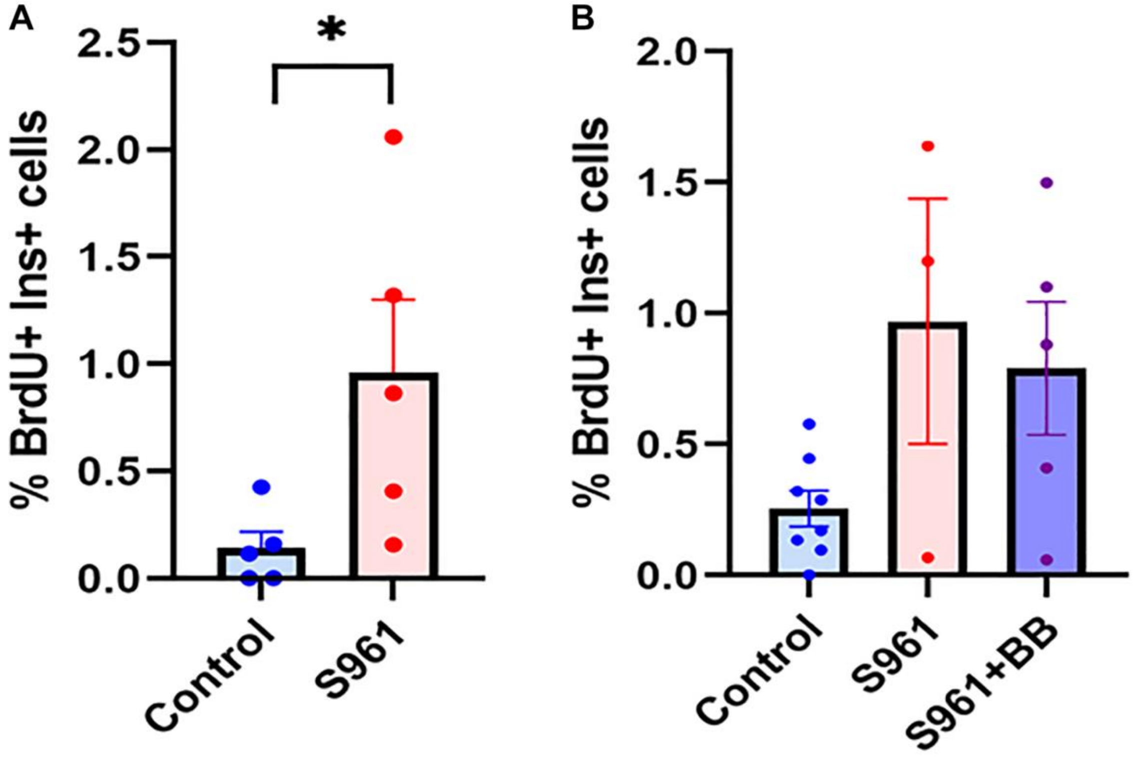

Supplementary Figure 6. (Related to Figure 5) (**A**) Proliferation of beta cells (%) in C57Bl6/J 8/9-month-old mice, $n_{control}$ = 5, n_{S961} = 5. (**B**) Proliferation of beta cells (%) in INK-ATTAC 18/19-month-old mice in all three groups.
